# Prediction of Mortality in Hemodialysis Patients Using Moving Multivariate Distance

**DOI:** 10.3389/fphys.2021.612494

**Published:** 2021-03-11

**Authors:** Mingxin Liu, Véronique Legault, Tamàs Fülöp, Anne-Marie Côté, Dominique Gravel, F. Guillaume Blanchet, Diana L. Leung, Sylvia Juhong Lee, Yuichi Nakazato, Alan A. Cohen

**Affiliations:** ^1^PRIMUS Research Group, Department of Family Medicine, University of Sherbrooke, Sherbrooke, QC, Canada; ^2^Research Center on Aging, Sherbrooke, QC, Canada; ^3^Department of Medicine, Geriatric Division, University of Sherbrooke, Sherbrooke, QC, Canada; ^4^Department of Medicine, Nephrology Division, University of Sherbrooke, Sherbrooke, QC, Canada; ^5^Research Center of Centre Hospitalier Universitaire de Sherbrooke, Sherbrooke, QC, Canada; ^6^Département de Biologie, Université de Sherbrooke, Sherbrooke, QC, Canada; ^7^Département de Mathématique, Université de Sherbrooke, Sherbrooke, QC, Canada; ^8^Department of Pathology, Yale University, New Haven, CT, United States; ^9^InfoCentre, Centre Intégré Universitaire de Santé et de Services Sociaux de l’Estrie – Centre Hospitalier Universitaire de Sherbrooke, Sherbrooke, QC, Canada; ^10^ Division of Nephrology, Yuai Nisshin Clinic, Hakuyukai Medical Corporation, Saitama, Japan

**Keywords:** network physiology, critical transition, early warning sign, multivariate statistical approaches, variability, early intervention

## Abstract

There is an increasingly widespread use of biomarkers in network physiology to evaluate an organism’s physiological state. A recent study showed that albumin variability increases before death in chronic hemodialysis patients. We hypothesized that a multivariate statistical approach would better allow us to capture signals of impending physiological collapse/death. We proposed a Moving Multivariate Distance (MMD), based on the Mahalanobis distance, to quantify the variability of the multivariate biomarker profile as a whole from one visit to the next. Biomarker profiles from a visit were used as the reference to calculate MMD at the subsequent visit. We selected 16 biomarkers (of which 11 are measured every 2 weeks) from blood samples of 763 chronic kidney disease patients hemodialyzed at the CHUS hospital in Quebec, who visited the hospital regularly (∼every 2 weeks) to perform routine blood tests. MMD tended to increase markedly preceding death, indicating an increasing intraindividual multivariate variability presaging a critical transition. In survival analysis, the hazard ratio between the 97.5th percentile and the 2.5th percentile of MMD reached as high as 21.1 [95% CI: 14.3, 31.2], showing that higher variability indicates substantially higher mortality risk. Multivariate approaches to early warning signs of critical transitions hold substantial clinical promise to identify early signs of critical transitions, such as risk of death in hemodialysis patients; future work should also explore whether the MMD approach works in other complex systems (i.e., ecosystems, economies), and should compare it to other multivariate approaches to quantify system variability.

## Introduction

Recent research on physiology increasingly shows that physiological systems do not operate independently. The coordination among different organs and systems maintains body homeodynamics; similarly, the proper functioning of each organ and system requires a healthy organism (Kennedy et al., 2014; Cheikhi et al., 2019; Franceschi et al., 2019). This is the purpose of network physiology, an emerging research field dedicated to the study of coordination among diverse systems at the organism level and dynamical transition of physiological states (Ivanov et al., 2016). Such coordination is critical; however, the dynamic complexity is challenging to untangle (Bashan et al., 2012). One approach to network physiology is to consider the organism as a single network of molecules (Cohen et al., 2012). While the structure of this network is far from being elucidated and many nodes are still unidentified, it may nonetheless be possible to infer much about the network via sampling of small subsets of nodes (i.e., molecules), due to the emergent properties of the network as a whole that lend coherence to its state and dynamics (Cohen, 2016; Cohen et al., 2021). This approach can be linked to a more general area of complex systems theory that has recently gained substantial attention: critical transitions (Dakos et al., 2008, 2012; Scheffer et al., 2009; Bashan et al., 2012; Ghalati et al., 2019). In complex systems, system dynamics such as a change in variability may provide early warning signs (EWSs) of impending state changes known as critical transitions (e.g., ecological collapse, financial crises, and shifts in climate regime). Only a few studies have explicitly applied this framework to health and disease (Gijzel et al., 2017; Ghalati et al., 2019; Nakazato et al., 2020), though many studies have reported higher variation in various biomarkers preceding the onset of adverse outcomes in subjects with chronic diseases (Holzel, 1987; Ma et al., 2012; Mendez et al., 2013; Myers et al., 2018; Woo et al., 2018), including Chronic Kidney Disease (CKD; Yang et al., 2007; Flythe et al., 2013; Selvarajah et al., 2014; Corte et al., 2015; Nakazato et al., 2017). Increased variance is indeed one of the main characteristics of resilience loss (Gijzel et al., 2017). Additionally, most studies of critical transitions evaluate univariate indices. A network physiology perspective implies coordination across systems in timing and dynamics, suggesting that a multivariate approach would allow better assessment of the coordinated physiological shifts.

One of the challenges in using clinical biomarker-based models of critical transitions in physiology is that detection of EWSs requires detailed time series in order to detect features such as increases in variance, autocorrelation, loss of resilience, etc. Chronic kidney disease (CKD) represents an excellent opportunity to circumvent this challenge: patients on hemodialysis are generally treated three times per week, with blood work conducted approximately every 2 weeks depending on local protocols. The health consequences of repeatedly missing visits can be severe, and treatment can continue for years or even decades. Accordingly, electronic medical records can provide time series with bi-weekly values for fixed biomarker panels spanning many years, with relatively few missing data for the core blood panels, and with little to no bias in terms of health state (i.e., the same markers are measured at the same time regardless of the presence or absence of other health problems). This is in contrast to cohort study data collected explicitly for research in humans, which rarely provide a dense enough time series, and to most other types of clinical data, where there are problems with the regularity of measurement, variation in the biomarkers measured, and a bias toward measurement only when health problems are suspected (i.e., sicker individuals).

Given the growing aging population, age-related chronic diseases like CKD are increasingly becoming a burden (O’Callaghan et al., 2011; Hill et al., 2016; Webster et al., 2017; Luyckx et al., 2018), and thus a prominent research topic. CKD does not solely consist of kidney dysfunction, but also leads to various systemic complications: cardiovascular disease and stroke, anemia, etc (Thomas et al., 2008; Webster et al., 2017). Usually, CKD-related complications, rather than CKD itself, lead to death (Muntner et al., 2002; Go et al., 2004; Nordio et al., 2012; Hill et al., 2016). Moreover, diabetes and hypertension, along with glomerulonephritis, are known to be the primary causes of CKD (Webster et al., 2017), stressing the importance of holistic approaches to age-related diseases (Kennedy et al., 2014). If EWSs were successfully assessed, early diagnosis and treatment adjustments could be performed in a timelier manner. In particular, patients with end-stage kidney disease (ESKD, the last stage of CKD) are often hospitalized, suffering substantial health comorbidities as individuals and representing an important burden on the health care system (Nordio et al., 2012; Webster et al., 2017; Luyckx et al., 2018). Hospitalizations and death may often represent what we term here “physiological collapse,” a critical transition in which the homeostatic/homeodynamic mechanisms are pushed outside the bounds they can properly respond to and thus require external intervention to maintain life. Early detection of impending physiological collapse events could provide the potential for less burdensome, less costly, and more effective interventions. For example, an algorithm to detect EWSs of physiological collapse could be built directly into electronic medical records systems, providing an alert when appropriate. Many dynamic signals of EWSs have been identified in critical transition literature more broadly: increased variability, decreased resilience, increased autocorrelations, increased cross-correlations, and critical slowing down. However, the accuracy and sensitivity of the establishment of such signals are still challenging (Scheffer et al., 2012), and medical applications are not well developed.

**FIGURE 1:**
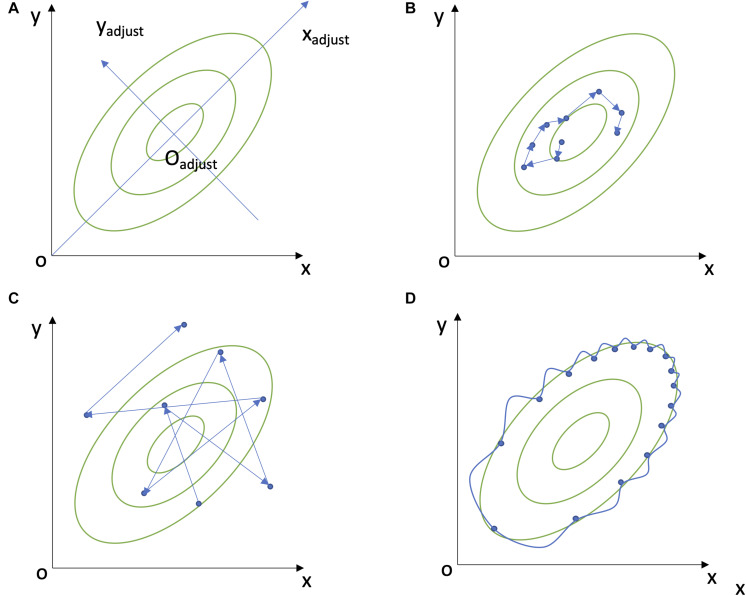
Explanations of Mahalanobis Distance and MMD. **(A)** A two-dimensional example of Mahalanobis distance. **(B)** A low dynamic movement of Moving Multivariate Distance (MMD), i.e., low intraindividual variability. **(C)** A high dynamic MMD movement, i.e., high intraindividual variability. **(D)** A two-dimensional autocorrelation.

Here, we focus on one EWS indicator: the increase in variability of a complex system, which is often linked to loss of resilience (Scheffer et al., 2012). Increase in variability reflects the longer time that the system with low resilience takes to recover from perturbations and return to an equilibrium state, a phenomenon named critical slowing down and which causes higher fluctuations (Scheffer et al., 2012; Kéfi et al., 2014). However, change in variability is still being studied one biomarker at a time (Ma et al., 2012; Flythe et al., 2013; Mendez et al., 2013; Nakazato et al., 2017; Woo et al., 2018), even though a multivariate signal would likely be more powerful. Within organisms, biomarkers are integrated into a complex physiological system in which levels of one depends on the levels of many other biomarkers; hence no single marker can truly reflect the underlying physiological state (Cohen et al., 2012; Cohen, 2016). Moreover, biomarkers can be sensitive to population composition, a problem that can be partially circumvented by integrating the interdependence of biomarkers into the equation (Cohen et al., 2017). More broadly, approaches to measure EWSs for critical transitions are generally univariate even though the systems in question (ecosystems, economies, etc.) are generally high-dimensional and interconnected. Multivariate EWSs are thus an important untapped field, and would link critical transition theory to network physiology, where synchronization across physiological systems is a major subject of interest. We have previously demonstrated the utility of statistical distance in measuring multivariate physiological dysregulation (PD) and predicting mortality, either at the organism level (Cohen et al., 2013, 2014, 2015; Milot et al., 2014) or in specific systems (Li et al., 2015). However, the previous work, based on Mahalanobis Distance (Mahalanobis, 1936), had only considered the deviation of biomarkers from an average population norm. Here, we hypothesized that intraindividual changes in biomarker variability could be captured with the same approach, but by calculating the distance of an individual’s multivariate position from that of the previous visit, instead of the distance from the population mean (Figure 1 and Supplementary Figure 1). We defined this measure as Moving Multivariate Distance (MMD) and tested it in a population of 763 patients with CKD under long-term hemodialysis. We hypothesized that intraindividual variability in biomarkers, as measured by MMD, would increase before a critical transition (in this case, death).

## Materials and Methods

### Dataset

Our study population consisted of 2565 patients who underwent hemodialysis from 1997 to 2017 at the *Centre Hospitalier Universitaire de Sherbrooke* (CHUS) in Quebec, Canada. Data were extracted from the CIRESSS platform, which aggregates all electronic hospital data for clinical and administrative purposes. Because the CHUS hospital system is the only tertiary hospital in the region, these data can be considered a nearly exhaustive representation of hemodialysis patients in the Eastern Townships region of Quebec (population ∼325K) for the period in question. From the 2565 patients, we excluded 1694 who were no longer treated by in-center hemodialysis at 6 months (potentially indicating death, recovery within 6 months and thus acute rather than chronic kidney failure, or transition to another renal replacement modality or to conservative care), and 58 patients with irregular hemodialysis visits and/or an acute or unspecified kidney failure diagnosis, leaving us with 813 patients having CKD and on long-term hemodialysis. For all the patients, we excluded the first 6 months on hemodialysis since dialysis initiation has been reported to represent a critical transition in itself (Broers et al., 2015), but for 26 patients, we were left with less than three blood sampling visits and thus excluded them. We also excluded 24 patients with incomplete biomarker data, yielding a total of 763 patients for analyses (Table 1). We define these 763 long-term hemodialysis patients as the “Full” dataset in the study, in contrast to the “Individuals 65+” dataset, those aged 65 years or more at their last data collection, from the 763 hemodialysis patients. However, some patients were lost to follow-up or had missing data (either they moved or stopped blood work due to palliative care) in the period just preceding their death. We expected that physiological signals would be strongest just prior to death, and thus created additional data subsets excluding individuals who did not have a visit within the last 30, 60, 90, 183, or 365 days preceding the date of death (loss to follow up, Supplementary Table 1). We also created a subset of “Kidney transplant” patients. As opposed to all censored patients which contain patients who were censored for unknown reasons or due to the end of the study period, which may happen not long before their death, this subset strictly contains patients censored by a kidney transplant. It was generated by selecting patients who had no hemodialysis visit and did not die in the 2 years following their kidney transplant. All trend plots only considered the last 5 years’ biomarker profiles for each patient (i.e., preceding death, kidney transplant, or loss to follow-up).

**TABLE 1:** Characteristics of study participants at first visit included in analyses (i.e., after excluding the first 6 months of dialysis).

**Characteristic**	**(*n* = 763)**
Age (years) ± SD	64.2 ± 15.8
Male, *n* (%)	479 (62.8)
Diabetic, *n* (%)	391 (51.2)
Death, *n* (%)	525 (68.8)
Hemodialysis time (years), median (IQR: 25%,75%)	2.12 (0.67, 4.44)
Received at least once successful kidney transplant* (%)	149 (19.5%)
Number of visits per patient, median (IQR: 25%,75%)	57 (21, 117)

*No subsequent dialysis 2 years after received a kidney transplant.

### Biomarker Selection

Patients under dialysis treatment have regular blood sampling, generally every 2 weeks; however, not all biomarkers are measured with the same frequency. We thus generated three blood schedule-based biomarker sets according to intervals at which they were available (Table 2). Therefore, the “Two weeks” biomarker set only includes the biomarkers that are tested every 2 weeks. The “One month” biomarker set includes the biomarkers that are tested every 2 weeks or every month. The “Four months” biomarker set includes all the biomarkers that we consider. Because the frequency of blood sampling was not perfect in our dataset, we considered all visits that occurred within 12 to 16 days as a two-week interval, 25 to 35 days as a one-month interval, and 100 to 140 days as a four-month interval. We excluded the following biomarkers because they were irregularly measured or had many missing values in our dataset: uric acid, ionized calcium, carbon dioxide, iron-binding capacity, iron, ferritin, iron saturation, transferrin, urea, glycated hemoglobin, partial carbon dioxide pressure, partial oxygen pressure, pH, intact parathyroid hormone, and thyroid-stimulating hormone.

**TABLE 2:** Biomarker information.

**Biomarker**	**Mean ± SD**	**Two weeks**	**One month**	**Four months**	**Physiological system**
Hematocrit (%)	0.33 ± 0.05	X	X	X	O_2_ transport
Hemoglobin (g/L)	107.72 ± 15.04	X	X	X	O_2_ transport
MCH* (pg)	31.44 ± 2.15	X	X	X	O_2_ transport
MCHC*(g/L)	329.70 ± 11.46	X	X	X	O_2_ transport
MCV* (fL)	95.33 ± 5.92	X	X	X	O_2_ transport
Platelet count (10^9^/L)	216.45 ± 84.94	X	X	X	–
Potassium (mmol/L)	4.76 ± 0.73	X	X	X	Kidney health
RBC count (10^12^/L)	3.44 ± 0.51	X	X	X	O_2_ transport
RDW (%)	15.75 ± 1.96	X	X	X	O_2_ transport
Sodium (mmol/L)	138.26 ± 3.78	X	X	X	Kidney health
WBC count (10^9^/L)	7.84 ± 3.66	X	X	X	–
Calcium (mmol/L)	2.23 ± 0.19		X	X	MBD**
Creatinine (μmol/L)	635.88 ± 291.58		X	X	Kidney health
Glucose (mmol/L)	7.63 ± 3.87		X	X	–
Phosphate (mmol/L)	1.53 ± 0.49		X	X	MBD**
Albumin (g/L)	36.27 ± 5.56			X	Kidney health

*The biomarkers are not mathematically independent from other selected biomarkers.

**MBD, mineral bone disease.

According to the physiological features of the biomarkers, we also classified them into three physiological systems (Table 2). Creatinine is commonly used to report estimated glomerular filtration rate (eGFR), which is the most important indicator for estimating general kidney function in clinical practice (Webster et al., 2017). Albuminuria, which implies continuous urinary loss, indicates pathological kidney damage which is caused by elevating membrane permeability (Webster et al., 2017). In addition, albumin level is decreased secondary to uremic state, chronic inflammation and malnutrition, which are present in ESKD, and is a marker of mortality (Zoccali et al., 2005; Honda et al., 2006; Phelan et al., 2008).Therefore, we classified these two biomarkers into the “Kidney health” group (Table 2). Impaired kidney function can also lead to electrolytic alteration (i.e., potassium derangement, dysnatremia, and dysmagnesemia; Dhondup and Qian, 2017). The reduced potassium excretory capacity, which usually causes hyperkalemia, has been shown to be significantly associated with hospitalization and the prognosis of CKD patients (Luo et al., 2016; Dhondup and Qian, 2017). Therefore, we also included potassium and sodium in the “Kidney health” group (Table 2). Kidney dysfunction could interfere with erythropoietin (EPO) production; this could further lead to anemia, which is one of the complications of CKD (Webster et al., 2017). Accordingly, we included seven biomarkers describing red blood cells in the “O_2_ transport” group (Table 2). Similarly, the kidney plays an essential role in regulating calcium and phosphate metabolism (Dhondup and Qian, 2017; Webster et al., 2017), and mineral bone disease is indeed one of CKD’s possible complication (Dhondup and Qian, 2017; Webster et al., 2017). We thus created a third group named “Mineral Bone Disease” which includes calcium and phosphate (Table 2). For each physiological group, we used the visit interval of the least frequently measured biomarker as the interval for MMD calculation (i.e., we use “Four months” as the visit interval for the “Kidney health” group since it includes “Albumin”). Lastly, MCH, MCHC, and MCV are mathematically redundant, since they can be calculated from hemoglobin, RBC count, and HCT. To check the effect of this redundancy on MMD, we conducted sensitivity analyses excluding the three redundant biomarkers from both the blood schedule-based biomarker sets and physiological system biomarker sets and then performed Cox proportional hazard models (see details in section “Survival Analysis”).

### Moving Multivariate Distance Calculation

We previously demonstrated that Mahalanobis distance can serve as a global measure of physiological dysregulation by calculating the distance of one’s biomarker profile relative to a population norm, essentially serving as a measure of aberrant physiological profile (Cohen et al., 2013, 2014, 2015; Milot et al., 2014; Li et al., 2015). In this previous work, we used a reference population (either the entire dataset or a younger and healthier population) to calculate the variance-covariance matrix (*S*) among biomarkers and mean values for each biomarker included in the Mahalanobis distance calculation (1). Since we were interested in measuring intraindividual rather than interindividual variation, here we used the individual biomarker profile of each previous visit *x*_*t–1*_ rather than the population mean *μ* as the reference for calculating the distance to its following biomarker profile *x*_*t*_ (Figures 1B,C), though the covariance among biomarkers *S* was still calculated at the population level (2).

(1) $$M\,a\,h\,a\,l\,a\,n\,o\,b\,i\,s\,D\,i\,s\,t\,a\,n\,c\,e=\sqrt{{\left(x-\mu \right)}^{T}\,S^{-1}\,\left(x-\mu \right)}$$

(2) $$M\,M\,D=\sqrt{{\left(x_{t}-x_{t-1}\right)}^{T}\,S^{-1}\,\left(x_{t}-x_{t-1}\right)}$$

Therefore, a higher MMD represents higher intraindividual multivariate variability. By using the previous state rather than the population mean as a reference population, and by using a time series, MMD measures something completely different than traditional Mahalanobis distance: the variability of an individual’s profile in physiological space (Supplementary Figure 1). However, *S*^–1^ is identical in Eqs 1, 2, calculated from all observations of *x* within each biomarker set, as it is assumed the physiological space defined by correlation structure is largely invariant across individuals.

The stationarity of the covariance matrix (=correlation matrix, since all variables are normalized) is a strong assumption in our model; future work will assess how the correlation structure may also evolve prior to critical transitions. Here, in order to test the importance of this assumption, we varied the variance-covariance matrices in three ways. First, we adjusted all the covariance values to 0 while keeping the variance (diagonal) in the variance-covariance matrix, *var-cov* (I). Then, we calculated the variance-covariance matrices by using only biomarker profiles from the last 3 months before death *var-cov* (II), and by using only those at least 2 years before death, *var-cov* (III).

Some biomarkers were log (white blood cell count, red cell distribution width, and glucose) or square-root (platelet count) transformed to better approach the assumption of multivariate normality in Mahalanobis distance, and all biomarkers were z-transformed according to the entire population mean and standard deviation before MMD calculation. We calculated the average among all the individual MMDs every half-year before death (or last contact for censored individuals, i.e., those who ceased hemodialysis after having a kidney transplant or were lost in follow-up before the end of the study) with 95% confidence intervals to visualize the MMD trend over time. Formal statistical tests of these trends are provided by the survival analysis in the next section. The R packages of “ggplot2”, “ggpubr” were used for visualization.

### Survival Analysis

To assess MMD’s predictive power of mortality, we ran Cox proportional hazards models with the package “survival (3.2-7)”, using years before death or last contact as the time-to-event variable. We controlled for sex and diabetic diagnosis, for age with a cubic spline (bs function, “fda” package), and for individuals using the cluster argument in the “coxph” function. We log-transformed MMD (log-MMD) for survival analysis since MMD is not normally distributed, and the calibration curve (“calibrate” function, “rms” package) of the log-transformed version showed a much more linear prediction in most cases, especially for the “Two weeks” biomarker set (data not shown). In a few cases, biomarker values at two consecutive visits yielded MMD equals to zero, which indicates no observed change in physiological condition from one visit to the next. In this case, we used half of the minimum value for the individual MMD, and then further performed log-transformation. We calculated the difference in hazard ratio (HR) between the 97.5th percentile and the 2.5th percentile (“HR95”) of log-MMD to illustrate the magnitude of the effect regardless of the scale of a continuous independent variable. We also checked whether the proportional hazards (PH) were constant over time, and thus that the model met the PH assumption by using the “cox.zph” function. We used the function “forestplot” for plotting. All analyses were run in R version 3.6.0. All code is available upon request.

## Results

Characteristics of our study population are shown in Table 1. Briefly, our study population was comprised of 763 patients on long-term hemodialysis (“Full” dataset) for an average of 3.3 years. 525 (68.8%) of the patients died before the end of the study, while the rest were censored (future outcome was unknown). One hundred forty-nine patients had at least once successful kidney transplant (i.e., no subsequent dialysis 2 years after having a kidney transplant). Half of the patients were diabetics (51.2%), nearly two thirds were men (62.8%). It should also be noted that, while the population had a large age range (16.2 to 94.6 years), more than half (67.8%). of the participants were aged 65 or older at their last visit included in analyses (“Individuals 65+” dataset).

### MMD Trends Over Time

**FIGURE 2:**
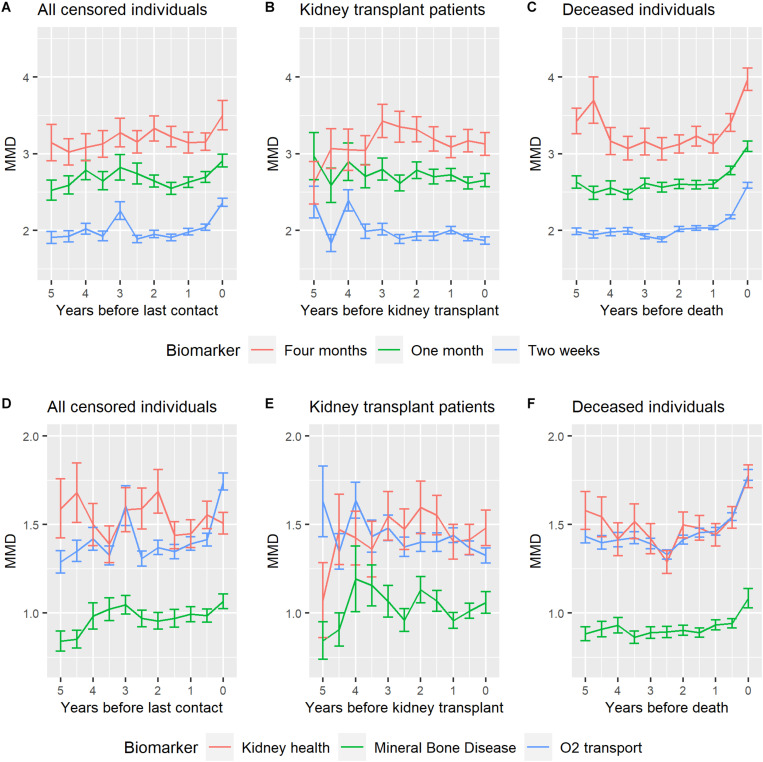
MMD half-year trends for different biomarker sets of hemodialysis patients. **(A,D)** show MMD of the censored individuals until their last contact. **(B,E)** show MMD of the individuals before receiving a successful kidney transplant (without subsequent dialysis 2 years after kidney transplant performed). **(C,F)** show MMD of the deceased individuals before the death, excluding individuals whose biomarker profile was missing during the last 30 days before the death. **(A–C)** used blood schedule-based biomarker sets, while **(D–F)** used physiological system biomarker sets. All the panels above were made based on the “Full” dataset (all the 763 hemodialysis patients). Note that the best statistical test for the differences observed here is the Cox models presented in Figure 3, Supplementary Figure 5, and Supplementary Tables 2–10. The trend graphics here are for illustrative purposes only.

Figure 2 shows MMD trends prior to last contact, kidney transplant, or death. For deceased individuals, the MMD of each three blood schedule-based biomarker sets shows an upward trend during the last year before death (Figure 2C). A similar but much less marked trend is visible for some analyses for the censored individuals (Figure 2A); however, no important trend is seen in biomarker sets for the individuals before receiving a kidney transplant (Figure 2B). Such trends were also evident when grouping the biomarkers by physiological system (Figures 2D–F). Such upward trends of the MMD prior to death were replicated regardless of whether individuals with missing data before death were excluded at different intervals (Supplementary Figures 3, 4), or the individuals’ age factor (Supplementary Figures 2, 4), or regardless of the covariance matrix used (Supplementary Figure 7).

### Survival Analysis

**FIGURE 3:**
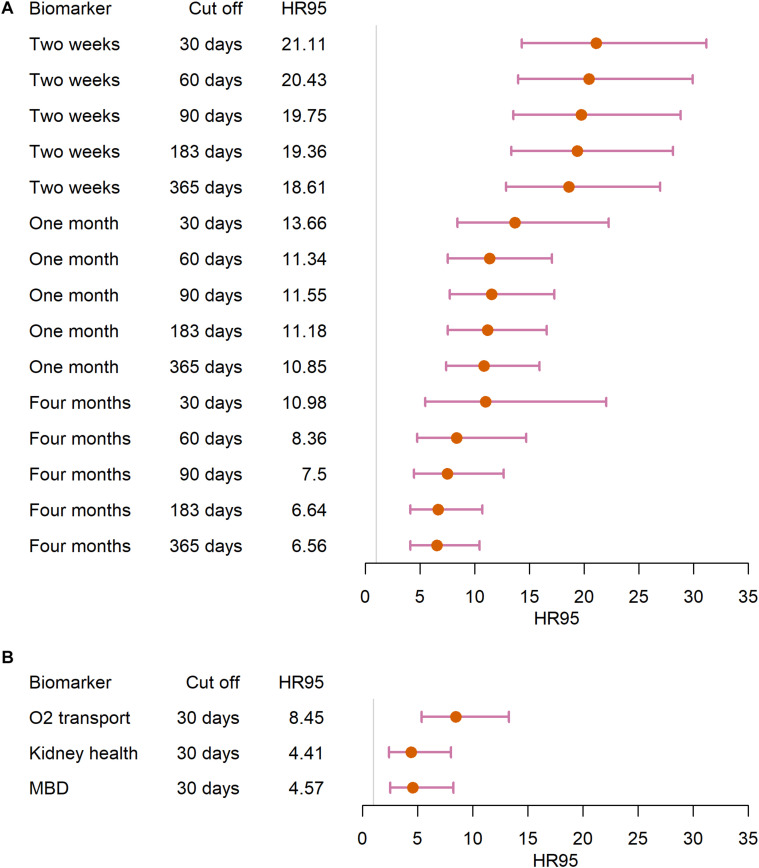
Survival analysis of the “Full” dataset. We ran Cox hazard proportional models in **(A)** the blood-schedule based biomarker sets and **(B)** the physiological system biomarker sets, using different cut offs (i.e., excluding individuals based on the time length of unavailable biomarker profiles). Points represent the difference in hazard ratio between the 97.5th percentile and the 2.5 percentile, and segments represent 95% confidence intervals.

MMD was found to be a strong predictor of mortality, regardless of detailed analytical decisions. Among the blood schedule-based biomarker sets, the “Two weeks” biomarker set gives the highest HR95 (Figure 3A). Generally speaking, signal increases slightly but not meaningfully as we are increasingly stringent about excluding individuals with missing visits prior to death (Figure 3A). Such results were replicated on the “Individuals 65+” dataset (Supplementary Figure 5). MMD in all three physiological systems was also associated with increased mortality risk (Figure 3B), though effects were generally much more modest than when combining all biomarkers. The O_2_ transport group gave the greatest HR95 among the physiological systems tested. Most Cox models were acceptable in terms of the PH assumption, though the PH assumption tended to be violated in models that did not exclude individuals missing data just prior to death (Supplementary Tables 2, 3 and 4). Results were also broadly replicated using different covariance matrices; results were somewhat stronger using an identity matrix for the variance (Supplementary Tables 7–9), and a bit weaker using the *var-cov* (II) (calculated based on individual’s biomarker profile from the last 3 months before death), but qualitative results are similar.

For the sensitivity analyses excluding the redundant biomarkers, results were qualitatively similar, and the “Two weeks” biomarker set and “O_2_ transport” physiological system biomarker set showed a higher HR95, indicating an even a stronger effect (Supplementary Tables 5, 6), however, both with a lower acceptance in terms of PH assumption (Supplementary Tables 5, 6). To test for any potential hidden bias in the data or the proposed methodology, notably a boundary effect where apparent but not real variability increases close to a data boundary, such as death, we calculated MMD by randomly shuffling visit order for each individual and found no evidence for such bias (Supplementary Figure 6 and details in Supplement).

## Discussion

Here, we defined the concept of MMD as a way to quantify the variability of an individual’s biomarker profile over time (or, more generally, the multivariate variability of a time series). MMD applies the concept of Mahalanobis distance, but rather than comparing a set of biomarkers to a population mean, it compares it from one visit to the subsequent one within one individual. A greater MMD thus indicates higher intraindividual multivariate variability. We showed that MMD increases markedly in the period preceding death in patients on hemodialysis, starting at 1 year before death but increasing exponentially in the few last months. Moreover, MMD is a strong predictor of mortality risk, and results were replicated using different sets of biomarkers based on their measurement frequency, with stronger effects using biomarker sets measured every 2 weeks compared to every month and every 4 months. These findings recapitulate the ones from Nakazato et al. (2017) showing greater changes in albumin variability in CKD patients, rather than changes in albumin levels per se (Nakazato et al., 2017). This finding was further supported with a similar approach but combining multiple biomarker coefficients of variation through principal components analysis (Nakazato et al., 2020).

From a network physiology perspective (Bashan et al., 2012; Ivanov et al., 2016), our findings confirm a synchronicity of variance increases prior to death across distinct physiological systems (Figures 2F, 3B). This synchronicity observed here appears largely due to changes in the variance rather than the covariance of markers, as the result is qualitatively similar when assessed with various covariance structures (Supplementary Figure 7). Future work will examine whether changes in the covariance structure might also be harnessed to improve predictions. Hemodialysis data such as we use here are not likely to be fine-scale enough to evaluate how signals propagate from one system to another, but other types of physiological data such as vital signs might permit this.

Additionally, mortality risk increases and the PH assumption is generally better respected as data become more complete prior to death, suggesting that we may be underestimating the true effects, or what might be detected with biomarker data at a finer time scale (Wen et al., 2018). Further validation in other medical conditions or datasets should be done to assess the sensitivity and efficiency of our approach. The poorer performance of specific physiological subsets compared to the full set of biomarkers suggests that signal increases substantially as more biomarkers are included, but this requires further validation and direct comparison. Also, as has been shown for clinical frailty (Fried et al., 2009; Ghachem et al., 2020), the additive dysfunction of many physiological systems might cause organismal collapse, rather than individual system dysfunction; hence a measure combining different physiological systems may better capture the underlying physiology and the synchronization of larger networks of systems. We also found an upward MMD trend in censored individuals in the few months preceding the end of the study period, though such trend appears later and is less obvious than for the deceased individuals. It probably reflects that all patients involved in the study were suffering from CKD and thus some, if not many, censored individuals were probably heading toward death. Another possible interpretation was that there could be a hidden bias in the data or the method that creates an artificial trend, but this was not supported by our results on shuffled visits (Supplementary Figure 6 and details in Supplement).

Various approaches have been developed to study critical transitions of complex systems in various domains. In neuroscience, Bashan et al. (2012) demonstrated that several integrated physiological systems play part in topological transition in sleep stages. Ghalati et al. (2019) used the Surprise Loss (SL) approach to characterize the critical transition that occurs before a septic shock. Dakos et al. (2008) illustrated the critical slowing down that precedes tipping points in climate transitions, using a time-series autocorrelation approach. Moreover, Dakos et al. (2012) compared different methods to predict critical transitions in ecological time series data, proposing a methodological guide that should be applicable in various fields. Nonetheless, none of the proposed approaches to critical transitions involved a multivariate description of the system.

In clinical practice, a reliable early diagnostic approach is in high demand since the current early diagnosis of chronic disease is still far from perfect (Webster et al., 2017). For CKD, many patients are asymptomatic and can only be detected by screening tests or at an advanced stage (Webster et al., 2017), which usually leads to a poor prognosis. Likewise, in patients with ESKD (GFR < 15 mL/min, per 1.73 m^2^, Webster et al., 2017), there are no current reliable indicators of physiological collapse. Management of CKD, particularly in older patients, remains a challenge, notably due to the interaction of CKD with other comorbidities (Anderson et al., 2009). Several mortality risk factors have a higher prevalence in CKD subjects, including lower physical activity level (Johansen et al., 2000), anemia (Astor et al., 2002; Ble et al., 2005), as well as cognitive decline and dementia (Seliger et al., 2004; Kurella et al., 2005; Hailpern et al., 2007). In this study, we have shown that the newly defined MMD approach can measure temporal intraindividual multivariate variability and may thus serve as an EWS in CKD patients. This model supposes a network physiology structure in which there is coordination across systems. The MMD approach can be used to quantify the global variability of multiple biomarkers, which indicate the dynamic from different physiological systems; thus, physiological network variability. Early prediction of underlying physiological change may help clinicians to manage these patients by indicating the need for further investigation or treatment. Before the system collapses, there are opportunities for the system to reverse to the equilibrium state or alternative stable states (Scheffer et al., 2001; Trefois et al., 2015).

There are also some limitations to this study. Time intervals in our study cohort were not always as precise as the prescribed blood test schedule, due to hospitalization events and other unknown reasons. To circumvent this problem, we only selected blood tests that felt into regular intervals but set a range of a few days to maximize the sample size. The smallest interval we used in the study was 2 weeks. However, a two-week interval is still a relatively long period from a physiological perspective, and the capacity to predict such a critical transition could be more powerful and precise if we could use a shorter interval (Wen et al., 2018). All patients in our study population were suffering from the same condition, even at the beginning of the study; thus, we did not compare our results with healthy participants, nor did we have records prior to the chronic kidney condition. Future studies should aim to make such comparisons. Lastly, we use a stationary covariance matrix for MMD calculation, a strong assumption in a dynamic network system. While results don’t change markedly with changes in the covariance matrix, future work will explore how changes in covariance might also be related to impending critical transitions.

Our multivariate approach shows promise for predicting critical transitions. Such detectable EWSs might prevent hospitalizations and complications, thereby saving lives and costs to the healthcare system by indicating the need for early interventions. Beyond CKD patients, our approach could also be applicable in other medical contexts (intensive care, congestive heart failure, cognitive decline, clinical frailty, or perhaps aging more generally), to predict non-adverse critical transitions (e.g., sleep and waking, or different sleep cycles), and even in other fields such as ecology (ecosystem collapses), economy (financial crises), and climate change. Thus, future work should validate our approach with other data and within other contexts and compare it to other methods for predicting critical transitions.

## Data Availability Statement

The datasets presented in this article are not readily available because, due to confidentiality concerns, transfer and sharing of individual-level data used in this study require prior approval from the Centre Informatisé de Recherche Évaluative en Services et Soins de Santé (CIRESSS) and the Director of Professional Services of the Centre Hospitalier Universitaire de Sherbrooke (CHUS), as well as by the Comité d’Éthique de la Recherche du CIUSSS de l’Estrie – CHUS. For this reason, raw data cannot be made publicly available. Requests to access the datasets should be directed to https://www.crchus.ca/en/services-outils/autres-services-et-outils/infocentre/.

## Ethics Statement

The studies involving human participants were reviewed and approved by Comité d’Éthique de la Recherche du CIUSSS de l’Estrie – CHUS. Written informed consent for participation was not required for this study in accordance with the National Legislation and the Institutional Requirements.

## Author Contributions

AAC, VL, ML, and DLL contributed to the study conception and design. SJL performed data collection. VL and DLL performed data preparation. ML, VL, DLL, and AAC performed data analysis. ML, VL, and AAC wrote the first draft of the manuscript. All authors commented on the manuscript, and read and approved the final manuscript.

## Conflict of Interest

AAC is Founder and CSO at Oken Health. The remaining authors declare that the research was conducted in the absence of any commercial or financial relationships that could be construed as a potential conflict of interest.
