# Supplementary material for: Prediction of Mortality in Hemodialysis Patients Using Moving Multivariate Distance
Source: Front Physiol. 2021 Mar 11;12:612494. doi: 10.3389/fphys.2021.612494 (PMC7993059; doi:10.3389/fphys.2021.612494)
Supplement: Supplementary file 1 [file Table_1.docx]

**Supplemental information for**

**Prediction of mortality in hemodialysis patients using moving multivariate distance**

Mingxin Liu^1^, Véronique Legault^1^, Tamàs Fülöp^2,3^, Anne-Marie Côté^4,5^, Dominique Gravel^6^, F. Guillaume Blanchet ^2,6,7^, Diana L. Leung^8^, Sylvia Juhong Lee^9^, Yuichi Nakazato^10^, and Alan A. Cohen^1^*

^1^ PRIMUS Research Group, Department of Family Medicine, University of Sherbrooke, Sherbrooke, Quebec, Canada

^2^ Research Center on Aging, Sherbrooke, Quebec, Canada

^3^ Department of Medicine, Geriatric Division, University of Sherbrooke, Sherbrooke, Quebec, Canada.

^4^ Department of Medicine, Nephrology Division, University of Sherbrooke, Sherbrooke, Quebec, Canada.

^5^ Research Center of Centre Hospitalier Universitaire de Sherbrooke

^6^ Département de biologie, Université de Sherbrooke, Sherbrooke, Quebec, Canada.

^7^Département de mathématique, Université de Sherbrooke, Sherbrooke, Québec, Canada.

^8^ Department of Pathology, Yale University, New Haven, CT, USA.

^9^ InfoCentre, Centre intégré universitaire de santé et de services sociaux de l’Estrie – Centre Hospitalier Universitaire de Sherbrooke, Sherbrooke, Quebec, Canada.

^10^ Division of Nephrology, Yuai Nisshin Clinic, Hakuyukai Medical Corporation, Saitama-City, Saitama, Japan

* Correspondence to: [Alan.Cohen@USherbrooke.ca](mailto:Alan.Cohen@USherbrooke.ca)

**Supplementary Methods**

**The potential boundary effect of MMD approach**

To justify the potential boundary effect of the MMD methodology, we randomly shuffled the order of the visits. In other words, within each individual, we took a full biomarker panel from a given visit and assigned it to a random visit from that individual, effectively changing the order without changing the timing or intervals of the visits, nor changing the coherence of a full biomarker profile from a given visit. We performed such calculations on both the "Full" dataset and the "Full" dataset excluding individuals with no visits during the last 30 days before death. Then we built the Cox proportional hazards model by using the log-transformed shuffled-MMD and the time series (Years before the death). For both trials, the HR95 of the shuffled-MMD was closely distributed around 1 (**Fig. S6**), with even maximal HR95 values well below the lower bounds of the confidence intervals in our least predictive unshuffled models, indicating no potential boundary effect of the MMD approach.

**The justification of using log transformed MMD in the Cox proportional hazard model.**

In addition to our principal analyses using log-MMD, we performed the Cox Proportional Hazard model by using the original MMD rather than the log transformation. It yields a much smaller HR95 but is still highly statistically significant and satisfies the proportional hazards assumption (**Table S10**). However, as indicated in the main text, the non-linearity of the MMD effect in the Cox model suggests that the log-MMD approach is preferable.

**Supplementary Tables**

**TABLE S1** | Numbers of individuals in each dataset

| **Dataset** | **Biomarker set** | **Cut off by missing follow-up till the death (Days)** | **Number of Individuals (Deceased Individuals)** |
| --- | --- | --- | --- |
| Full | Two weeks | 30 | 651(413) |
|  |  | 60 | 665(427) |
|  |  | 90 | 672(434) |
|  |  | 183 | 681(443) |
|  |  | 365 | 695(457) |
|  | One month | 30 | 567(338) |
|  |  | 60 | 633(404) |
|  |  | 90 | 646(417) |
|  |  | 183 | 653(424) |
|  |  | 365 | 674(445) |
|  | Four months | 30 | 421(197) |
|  |  | 60 | 517(293) |
|  |  | 90 | 572(348) |
|  |  | 183 | 626(402) |
|  |  | 365 | 657(433) |
| Individuals | Two weeks | 30 | 441(342) |
| 65+ |  | 60 | 451(352) |
|  |  | 90 | 455(356) |
|  |  | 183 | 463(364) |
|  |  | 365 | 474(375) |
|  | One month | 30 | 368(275) |
|  |  | 60 | 426(333) |
|  |  | 90 | 434(341) |
|  |  | 183 | 441(348) |
|  |  | 365 | 459(366) |
|  | Four months | 30 | 250(158) |
|  |  | 60 | 328(236) |
|  |  | 90 | 375(283) |
|  |  | 183 | 424(332) |
|  |  | 365 | 449(357) |

**TABLE S2** | Survival Analysis on the "Full" dataset

| Biomarker  set | Cut off by missing follow up till the death (Days) | LCI | HR95 | UCI | P-Value | PH Assumption  (log-MMD  P-Value) | PH Assumption  (Global  P-Value) |
| --- | --- | --- | --- | --- | --- | --- | --- |
| Two | 30 | 14.30 | 21.11 | 31.16 | <0.0001 | 0.0135 | 0.0589 |
| weeks | 60 | 13.96 | 20.43 | 29.90 | <0.0001 | 0.0135 | 0.0829 |
|  | 90 | 13.54 | 19.75 | 28.80 | <0.0001 | 0.0046 | 0.0428 |
|  | 183 | 13.33 | 19.36 | 28.12 | <0.0001 | 0.0021 | 0.0327 |
|  | 365 | 12.85 | 18.61 | 26.94 | <0.0001 | 0.0004 | 0.0063 |
| One | 30 | 8.40 | 13.66 | 22.22 | <0.0001 | 0.2851 | 0.6355 |
| month | 60 | 7.54 | 11.34 | 17.06 | <0.0001 | 0.1562 | 0.0778 |
|  | 90 | 7.72 | 11.55 | 17.27 | <0.0001 | 0.1716 | 0.0203 |
|  | 183 | 7.53 | 11.18 | 16.58 | <0.0001 | 0.0620 | 0.0050 |
|  | 365 | 7.39 | 10.85 | 15.93 | <0.0001 | 0.0676 | 0.0012 |
| Four | 30 | 5.47 | 10.98 | 22.02 | <0.0001 | 0.4051 | 0.9194 |
| months | 60 | 4.76 | 8.36 | 14.69 | <0.0001 | 0.3288 | 0.5552 |
|  | 90 | 4.45 | 7.50 | 12.65 | <0.0001 | 0.1031 | 0.2181 |
|  | 183 | 4.12 | 6.64 | 10.70 | <0.0001 | 0.0662 | 0.0803 |
|  | 365 | 4.12 | 6.56 | 10.45 | <0.0001 | 0.1033 | 0.4928 |

**TABLE S3** | Survival Analysis on "Individuals 65+" dataset

| Biomarker  set | Cut off by missing follow up till the death (Days) | LCI | HR95 | UCI | P-Value | PH Assumption  (log-MMD  P-Value) | PH Assumption  (Global  P-Value) |
| --- | --- | --- | --- | --- | --- | --- | --- |
| Two | 30 | 13.35 | 21.01 | 33.08 | <0.0001 | 0.0001 | 0.0004 |
| weeks | 60 | 13.03 | 20.30 | 31.63 | <0.0001 | 0.0004 | 0.0010 |
|  | 90 | 12.86 | 20.00 | 31.11 | <0.0001 | 0.0002 | 0.0016 |
|  | 183 | 12.51 | 19.37 | 29.98 | <0.0001 | 0.0000 | 0.0012 |
|  | 365 | 11.82 | 18.27 | 28.24 | <0.0001 | 0.0000 | 0.0002 |
| One | 30 | 7.04 | 11.92 | 20.20 | <0.0001 | 0.6889 | 0.2475 |
| month | 60 | 6.22 | 9.61 | 14.85 | <0.0001 | 0.7988 | 0.0820 |
|  | 90 | 6.42 | 9.90 | 15.29 | <0.0001 | 0.8603 | 0.0160 |
|  | 183 | 6.32 | 9.67 | 14.79 | <0.0001 | 0.5125 | 0.0054 |
|  | 365 | 6.19 | 9.38 | 14.22 | <0.0001 | 0.6150 | 0.0047 |
| Four | 30 | 5.31 | 12.01 | 27.17 | <0.0001 | 0.7729 | 0.6532 |
| months | 60 | 4.74 | 9.10 | 17.46 | <0.0001 | 0.7140 | 0.4565 |
|  | 90 | 4.22 | 7.60 | 13.68 | <0.0001 | 0.3643 | 0.0941 |
|  | 183 | 3.87 | 6.63 | 11.33 | <0.0001 | 0.2312 | 0.0476 |
|  | 365 | 3.72 | 6.27 | 10.56 | <0.0001 | 0.1897 | 0.2921 |

**TABLE S4**| Survival Analysis of physiological system biomarker sets on "Full" dataset

| Biomarker  set | Cut off by missing follow up till the death (Days) | LCI | HR95 | UCI | P-Value | PH Assumption  (log-MMD  P-Value) | PH Assumption  (Global  P-Value) |
| --- | --- | --- | --- | --- | --- | --- | --- |
| O_2_ transport | 30 | 5.38 | 8.45 | 13.27 | <0.0001 | 0.0242 | 0.1001 |
| Kidney health | 30 | 2.42 | 4.41 | 8.04 | <0.0001 | 0.3411 | 0.3143 |
| Mineral Bone Disease | 30 | 2.53 | 4.57 | 8.24 | <0.0001 | 0.8847 | 0.6595 |

**TABLE S5**| Survival Analysis on the "Full" dataset excluding redundant biomarkers (MCH, MCHC, and MCV).

| Biomarker  set | Cut off by missing follow up till the death (Days) | LCI | HR95 | UCI | P-Value | PH Assumption  (log-MMD  P-Value) | PH Assumption  (Global  P-Value) |
| --- | --- | --- | --- | --- | --- | --- | --- |
| Two | 30 | 21.33 | 32.32 | 48.97 | <0.0001 | 0.0132 | 0.0228 |
| weeks | 60 | 20.81 | 31.34 | 47.19 | <0.0001 | 0.0145 | 0.0409 |
|  | 90 | 20.24 | 30.42 | 45.72 | <0.0001 | 0.0058 | 0.0204 |
|  | 183 | 19.51 | 29.26 | 43.89 | <0.0001 | 0.0022 | 0.0167 |
|  | 365 | 18.41 | 27.58 | 41.29 | <0.0001 | 0.0003 | 0.0028 |
| One | 30 | 10.52 | 16.79 | 26.82 | <0.0001 | 0.0308 | 0.2812 |
| month | 60 | 8.46 | 12.89 | 19.62 | <0.0001 | 0.0233 | 0.0360 |
|  | 90 | 8.67 | 13.1 | 19.79 | <0.0001 | 0.0299 | 0.0125 |
|  | 183 | 8.18 | 12.33 | 18.59 | <0.0001 | 0.0058 | 0.0019 |
|  | 365 | 7.86 | 11.75 | 17.57 | <0.0001 | 0.0031 | 0.0002 |
| Four | 30 | 4.74 | 10.01 | 21.16 | <0.0001 | 0.1752 | 0.5248 |
| months | 60 | 4.71 | 8.44 | 15.11 | <0.0001 | 0.1308 | 0.2530 |
|  | 90 | 4.6 | 7.8 | 13.21 | <0.0001 | 0.0334 | 0.0891 |
|  | 183 | 4.32 | 6.99 | 11.29 | <0.0001 | 0.0307 | 0.0444 |
|  | 365 | 4.26 | 6.82 | 10.91 | <0.0001 | 0.0355 | 0.2878 |

**TABLE S6**| Survival Analysis of physiological system biomarker sets on "Full dataset" excluding redundant biomarkers (MCH, MCHC, and MCV).

| Biomarker  set | Cut off by missing follow up till the death (Days) | LCI | HR95 | UCI | P-Value | PH Assumption  (log-MMD  P-Value) | PH Assumption  (Global  P-Value) |
| --- | --- | --- | --- | --- | --- | --- | --- |
| O_2_ transport | 30 | 7.38 | 11.87 | 19.08 | <0.0001 | 0.0088 | 0.0362 |
| Kidney health | 30 | 2.42 | 4.41 | 8.04 | <0.0001 | 0.3411 | 0.3143 |
| Mineral Bone Disease | 30 | 2.53 | 4.57 | 8.24 | <0.0001 | 0.8847 | 0.6595 |

**TABLE S7** | Survival Analysis on the "Full dataset", using *var-cov* (I)

| Biomarker  set | Cut off by missing follow up till the death (Days) | LCI | HR95 | UCI | P-Value | PH Assumption  (log-MMD  P-Value) | PH Assumption  (Global  P-Value) |
| --- | --- | --- | --- | --- | --- | --- | --- |
| Two | 30 | 22.46 | 33.75 | 50.72 | <0.0001 | 0.0231 | 0.0659 |
| weeks | 60 | 21.68 | 32.43 | 48.52 | <0.0001 | 0.0221 | 0.0900 |
|  | 90 | 21.40 | 31.97 | 47.76 | <0.0001 | 0.0111 | 0.0586 |
|  | 183 | 20.22 | 30.25 | 45.25 | <0.0001 | 0.0026 | 0.0311 |
|  | 365 | 18.81 | 28.20 | 42.27 | <0.0001 | 0.0003 | 0.0025 |
| One | 30 | 11.60 | 18.28 | 28.82 | <0.0001 | 0.0282 | 0.2412 |
| month | 60 | 9.17 | 13.79 | 20.75 | <0.0001 | 0.0201 | 0.0262 |
|  | 90 | 9.33 | 13.93 | 20.81 | <0.0001 | 0.0252 | 0.0073 |
|  | 183 | 8.83 | 13.20 | 19.73 | <0.0001 | 0.0044 | 0.0008 |
|  | 365 | 8.39 | 12.48 | 18.56 | <0.0001 | 0.0014 | 0.0000 |
| Four | 30 | 4.72 | 9.80 | 20.37 | <0.0001 | 0.3188 | 0.8379 |
| months | 60 | 4.55 | 8.03 | 14.19 | <0.0001 | 0.2456 | 0.4441 |
|  | 90 | 4.61 | 7.64 | 12.67 | <0.0001 | 0.0912 | 0.2313 |
|  | 183 | 4.29 | 6.78 | 10.73 | <0.0001 | 0.0673 | 0.0862 |
|  | 365 | 4.10 | 6.43 | 10.09 | <0.0001 | 0.0403 | 0.3428 |

**TABLE S8** | Survival Analysis on the "Full dataset", using *var-cov* (II)

| Biomarker  set | Cut off by missing follow up till the death (Days) | LCI | HR95 | UCI | P-Value | PH Assumption  (log-MMD  P-Value) | PH Assumption  (Global  P-Value) |
| --- | --- | --- | --- | --- | --- | --- | --- |
| Two | 30 | 10.89 | 15.96 | 23.38 | <0.0001 | 0.0240 | 0.0854 |
| weeks | 60 | 10.70 | 15.55 | 22.60 | <0.0001 | 0.0239 | 0.1162 |
|  | 90 | 10.49 | 15.19 | 21.99 | <0.0001 | 0.0105 | 0.0683 |
|  | 183 | 10.29 | 14.85 | 21.41 | <0.0001 | 0.0049 | 0.0502 |
|  | 365 | 9.88 | 14.22 | 20.45 | <0.0001 | 0.0010 | 0.0101 |
| One | 30 | 7.16 | 11.49 | 18.43 | <0.0001 | 0.3008 | 0.6491 |
| month | 60 | 6.39 | 9.49 | 14.10 | <0.0001 | 0.1599 | 0.0694 |
|  | 90 | 6.52 | 9.62 | 14.20 | <0.0001 | 0.1795 | 0.0182 |
|  | 183 | 6.40 | 9.38 | 13.75 | <0.0001 | 0.0777 | 0.0047 |
|  | 365 | 6.39 | 9.30 | 13.55 | <0.0001 | 0.1067 | 0.0012 |
| Four | 30 | 4.97 | 9.82 | 19.39 | <0.0001 | 0.4575 | 0.9643 |
| months | 60 | 4.34 | 7.63 | 13.39 | <0.0001 | 0.4064 | 0.6597 |
|  | 90 | 4.04 | 6.81 | 11.48 | <0.0001 | 0.1422 | 0.2871 |
|  | 183 | 3.66 | 5.89 | 9.48 | <0.0001 | 0.0939 | 0.1016 |
|  | 365 | 3.65 | 5.81 | 9.24 | <0.0001 | 0.1329 | 0.5594 |

**TABLE S9** | Survival Analysis on the "Full dataset", using *var-cov* (III)

| Biomarker  set | Cut off by missing follow up till the death (Days) | LCI | HR95 | UCI | P-Value | PH Assumption  (log-MMD  P-Value) | PH Assumption  (Global  P-Value) |
| --- | --- | --- | --- | --- | --- | --- | --- |
| Two | 30 | 13.29 | 19.57 | 28.83 | <0.0001 | 0.0095 | 0.0527 |
| weeks | 60 | 12.95 | 18.90 | 27.60 | <0.0001 | 0.0092 | 0.0724 |
|  | 90 | 12.54 | 18.24 | 26.54 | <0.0001 | 0.0028 | 0.0340 |
|  | 183 | 12.41 | 17.98 | 26.06 | <0.0001 | 0.0014 | 0.0261 |
|  | 365 | 12.10 | 17.47 | 25.22 | <0.0001 | 0.0004 | 0.0058 |
| One | 30 | 7.79 | 12.73 | 20.80 | <0.0001 | 0.3845 | 0.6810 |
| month | 60 | 7.12 | 10.73 | 16.18 | <0.0001 | 0.1985 | 0.0830 |
|  | 90 | 7.33 | 11.00 | 16.50 | <0.0001 | 0.2198 | 0.0209 |
|  | 183 | 7.20 | 10.72 | 15.97 | <0.0001 | 0.0891 | 0.0056 |
|  | 365 | 7.10 | 10.45 | 15.40 | <0.0001 | 0.1069 | 0.0015 |
| Four | 30 | 5.21 | 10.42 | 20.83 | <0.0001 | 0.4366 | 0.9504 |
| months | 60 | 4.65 | 8.14 | 14.23 | <0.0001 | 0.3520 | 0.6195 |
|  | 90 | 4.29 | 7.18 | 12.03 | <0.0001 | 0.1231 | 0.2672 |
|  | 183 | 3.98 | 6.39 | 10.24 | <0.0001 | 0.0811 | 0.0997 |
|  | 365 | 4.01 | 6.35 | 10.06 | <0.0001 | 0.1389 | 0.5492 |

**TABLE S10** | Survival Analysis on the "Full dataset", while using MMD (rather than the log transformed)

| Biomarker  set | Cut off by missing follow up till the death (Days) | LCI | HR95 | UCI | P-Value | PH Assumption  (log-MMD  P-Value) | PH Assumption  (Global  P-Value) |
| --- | --- | --- | --- | --- | --- | --- | --- |
| Two | 30 | 1.86 | 2.29 | 2.81 | <0.0001 | 0.4454 | 0.3006 |
| weeks | 60 | 1.86 | 2.28 | 2.79 | <0.0001 | 0.3462 | 0.3322 |
|  | 90 | 1.85 | 2.26 | 2.75 | <0.0001 | 0.1944 | 0.2276 |
|  | 183 | 1.86 | 2.26 | 2.75 | <0.0001 | 0.2183 | 0.2366 |
|  | 365 | 1.86 | 2.26 | 2.75 | <0.0001 | 0.2013 | 0.1594 |
| One | 30 | 1.67 | 2.20 | 2.89 | <0.0001 | 0.9723 | 0.7646 |
| month | 60 | 1.70 | 2.24 | 2.94 | <0.0001 | 0.5839 | 0.0755 |
|  | 90 | 1.71 | 2.25 | 2.96 | <0.0001 | 0.3958 | 0.0378 |
|  | 183 | 1.72 | 2.26 | 2.97 | <0.0001 | 0.6190 | 0.0158 |
|  | 365 | 1.73 | 2.27 | 2.98 | <0.0001 | 0.3780 | 0.0019 |
| Four | 30 | 2.36 | 4.19 | 7.46 | <0.0001 | 0.8383 | 0.9965 |
| months | 60 | 2.46 | 3.62 | 5.34 | <0.0001 | 0.9511 | 0.8882 |
|  | 90 | 2.62 | 3.72 | 5.29 | <0.0001 | 0.6087 | 0.6210 |
|  | 183 | 2.62 | 3.55 | 4.81 | <0.0001 | 0.4608 | 0.3104 |
|  | 365 | 2.64 | 3.53 | 4.71 | <0.0001 | 0.6466 | 0.8837 |

**Supplementary Figures**

**
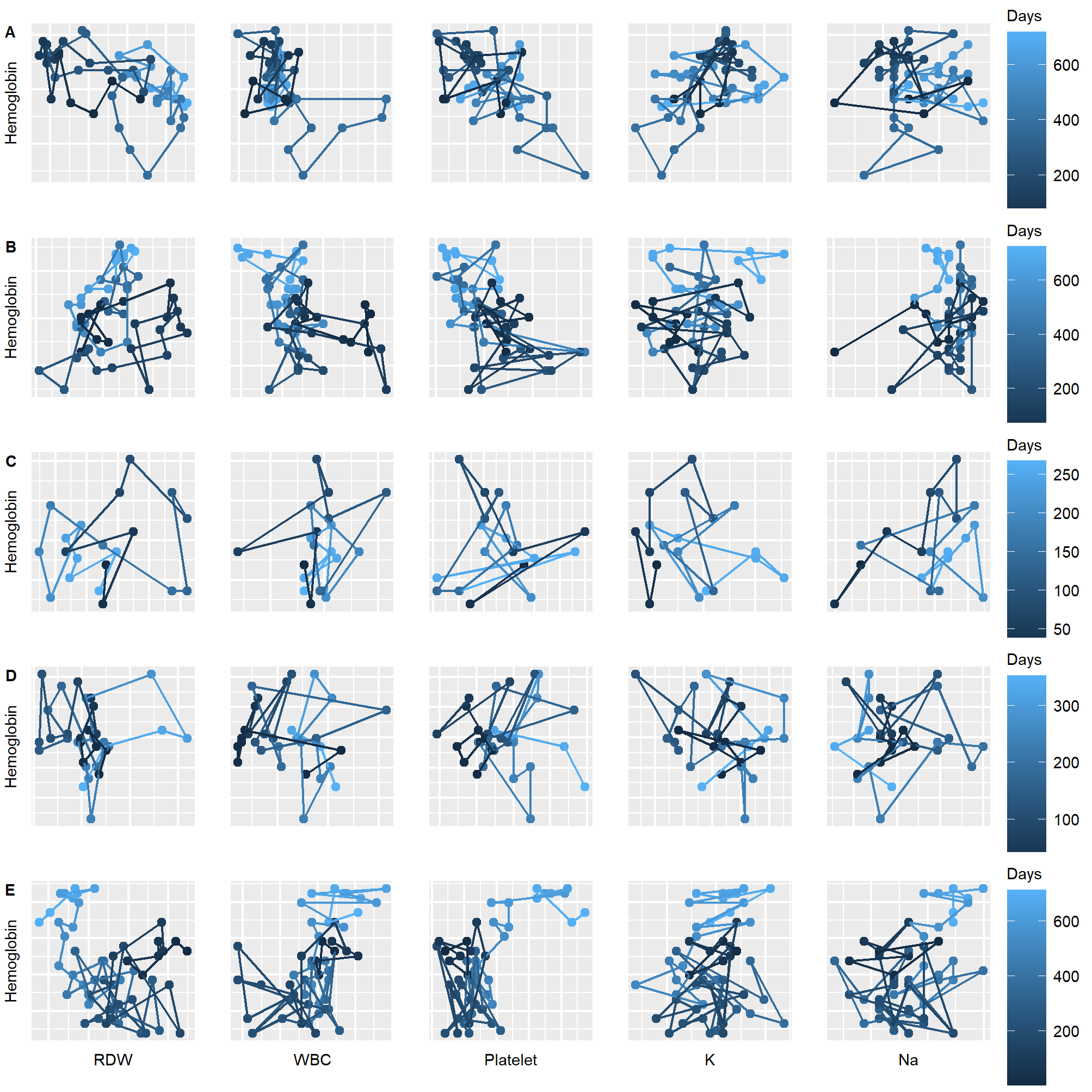
**

**Figure S1 Physiological dynamics of an individual's profile in physiological space.**

A), B), C), D), E) are five individuals that were randomly selected from the "Full" dataset with final visit data less than 30 days before the death. Panels show hemoglobin (y-axis) in relation to five additional biomarkers (x-axes, columns). The dot represents an individual physiological state at a given time, moving from light to dark blue as death approaches. The paths show consecutive variability of the time series. **"**Days" represents the days before the death. The two variables in all the biomarker pairs are not mathematically dependent. Scales are optimized in each panel to maximize the variability shown, and are thus not comparable across individuals.

**
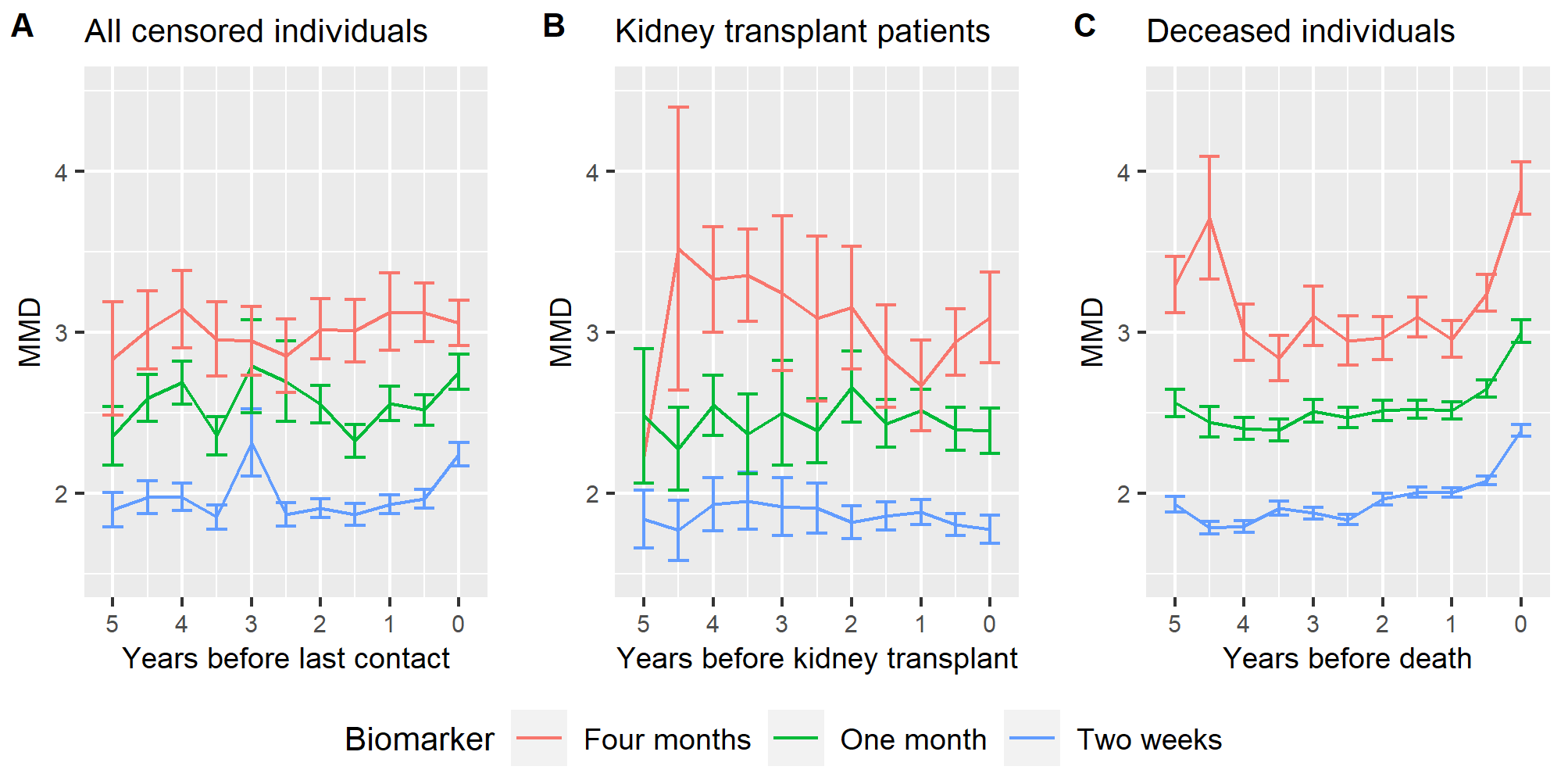
**

**Figure S2 MMD half-year trends of blood schedule-based biomarkers on** "**Individuals 65+**" **dataset.** A) MMD of censored individuals till their last contact. B)* MMD of the individuals who received a successful kidney transplant (without subsequent dialysis two years after kidney transplant performed). C) MMD of deceased individuals before their death, excluding individuals whose biomarker profile was missing during the last 30 days before the death.

*There are only one pair of “Four months” blood panel observations available to calculate MMD at five years before the kidney transplant.

**
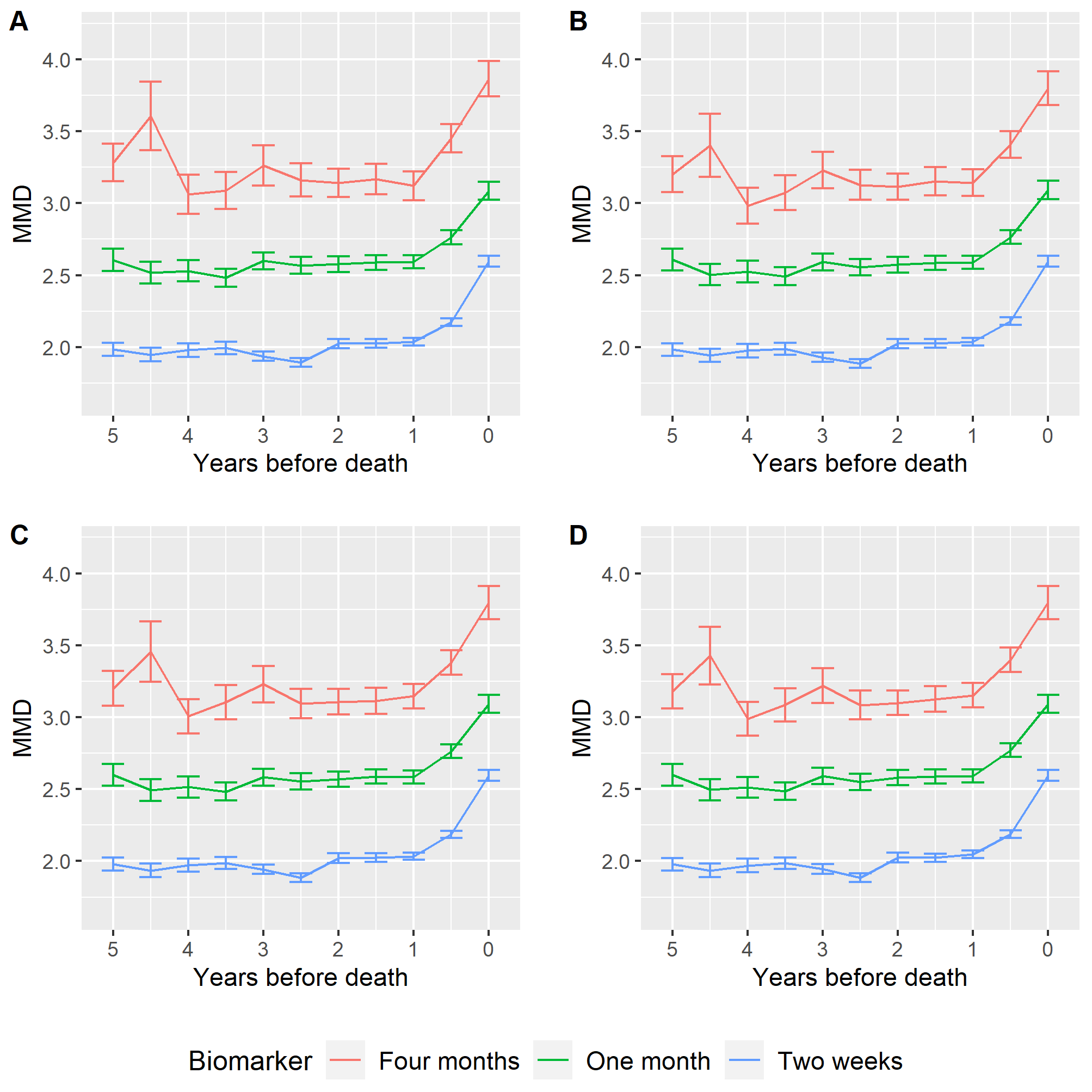
**

**Figure S3 MMD yearly trends of the deceased individual on the "Full" dataset, based on proximity of the last biomarker profile to death.** A) last visit within 60 days. B) last visit within 90 days. C) last visit within 183 days. D) last visit within one year.

**
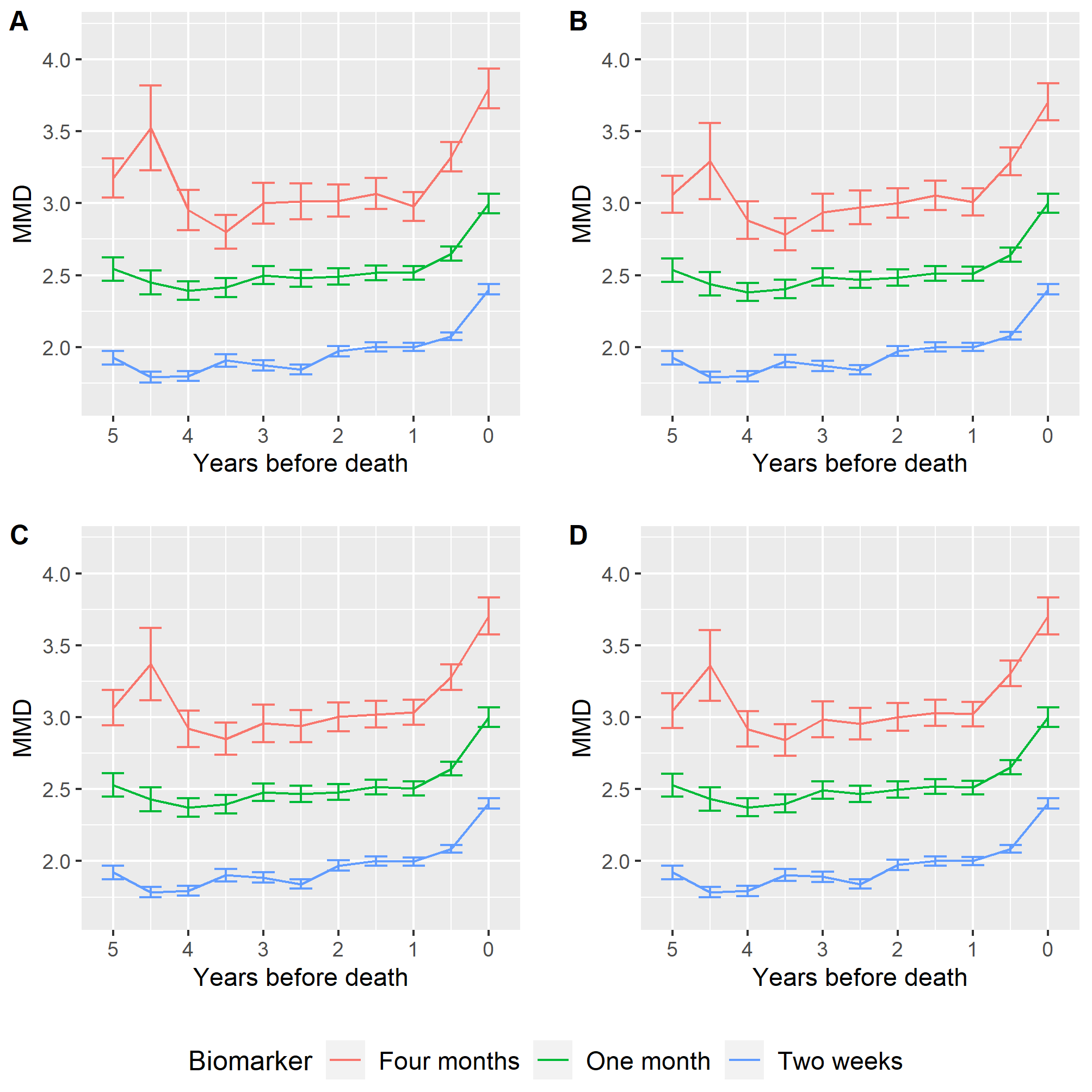
**

**Figure S4 MMD yearly trends of the deceased individual on "Individuals 65+" dataset, based on proximity of the last biomarker profile to death.** A) last visit within 60 days. B) last visit within 90 days. C) last visit within 183 days. D) last visit within one year.

**
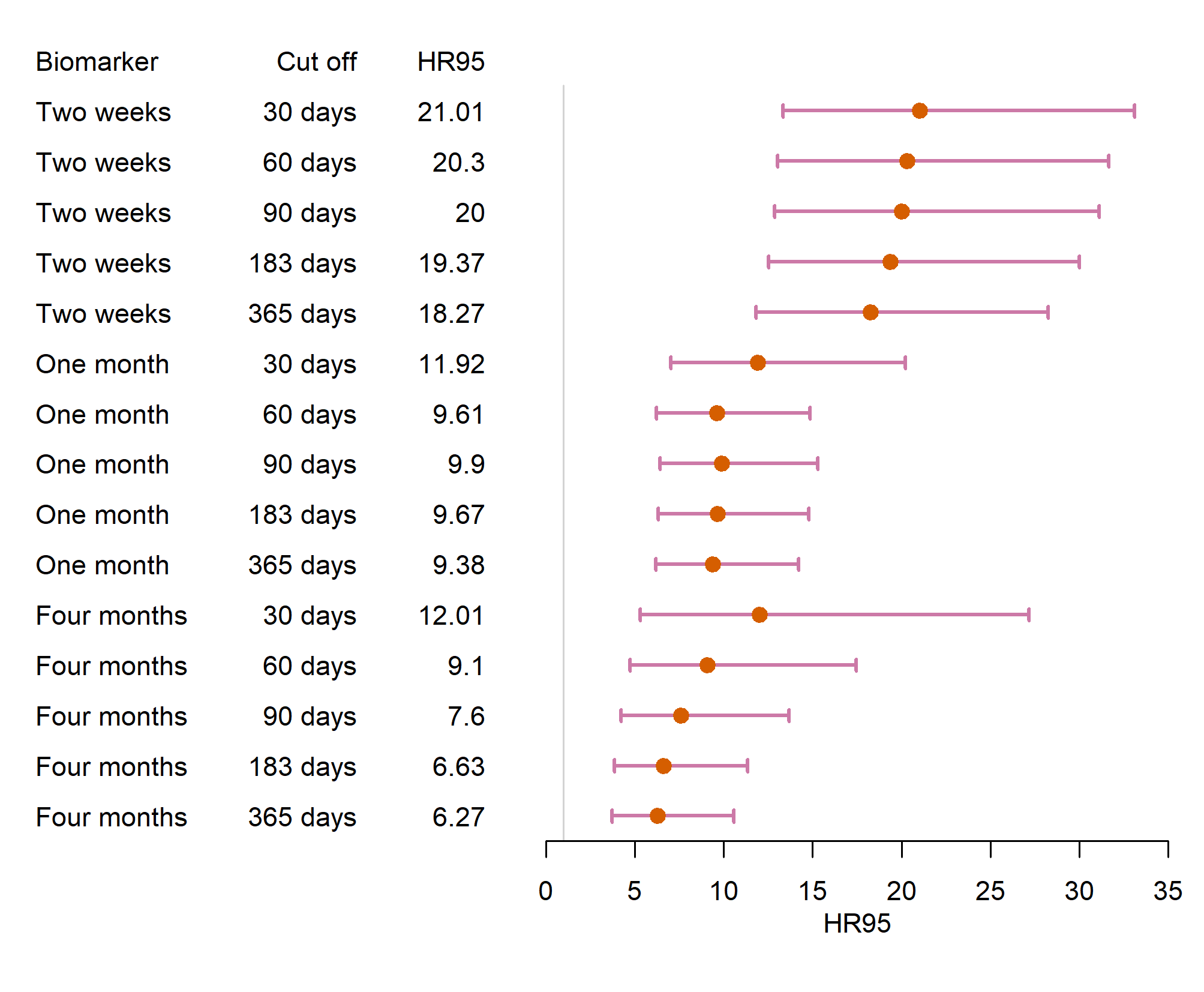
**

**Figure S5** **Survival analysis of the "Individuals 65+" dataset of blood schedule-based biomarker sets.** We ran Cox hazard proportional models in the blood-schedule based biomarker sets using different cut offs (i.e. excluding individuals based on the time length of unavailable biomarker profiles). Points represent the difference in hazard ratio between the 97.5^th^ percentile and the 2.5 percentile, and segments represent 95% confidence intervals.


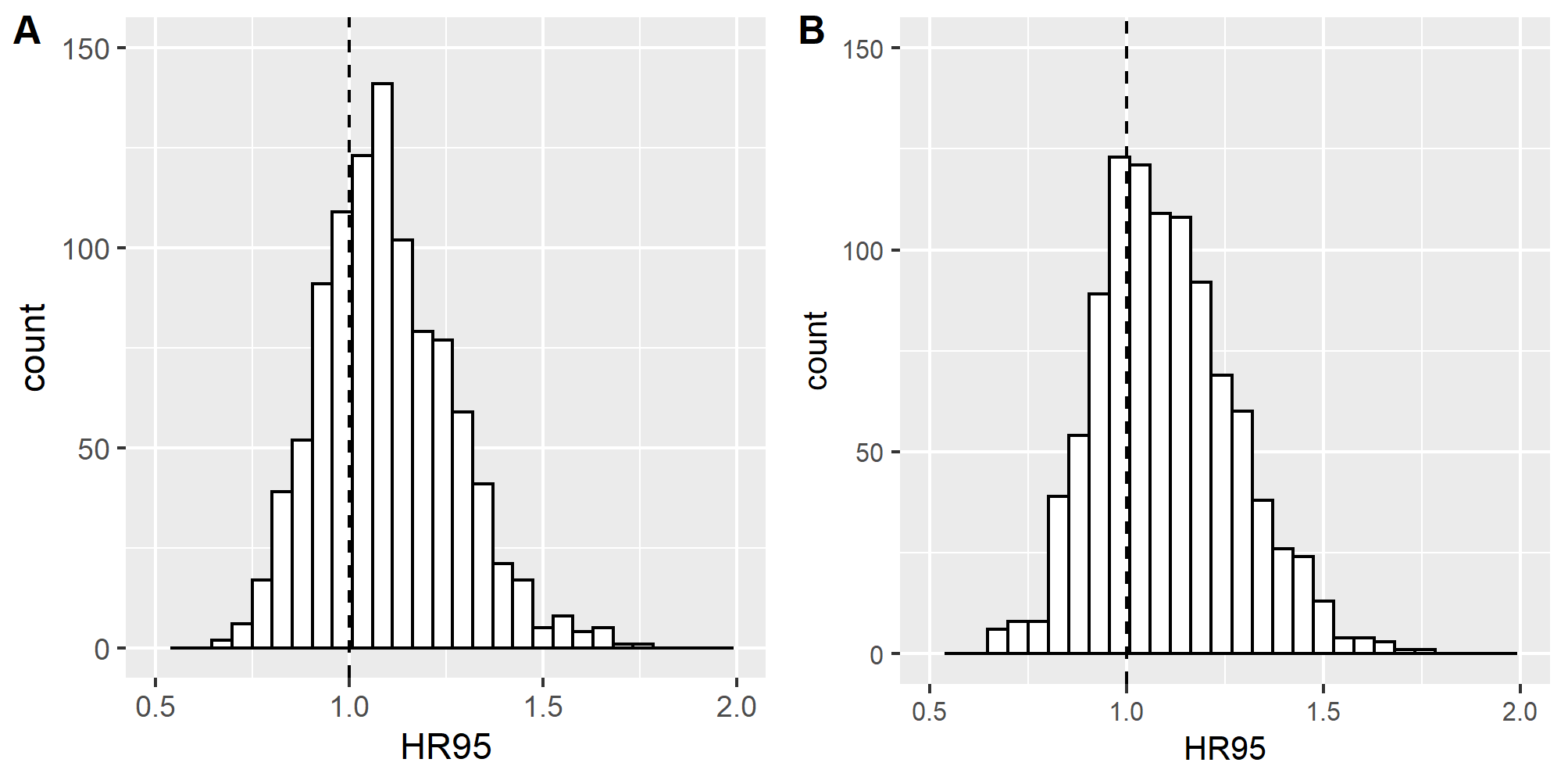
**Figure S6. Distribution of HR95 on the shuffled MMD** A) Analysis on "Full" dataset B) Analysis on "Full" data collection with data biomarker profile less than 30 days before the death.


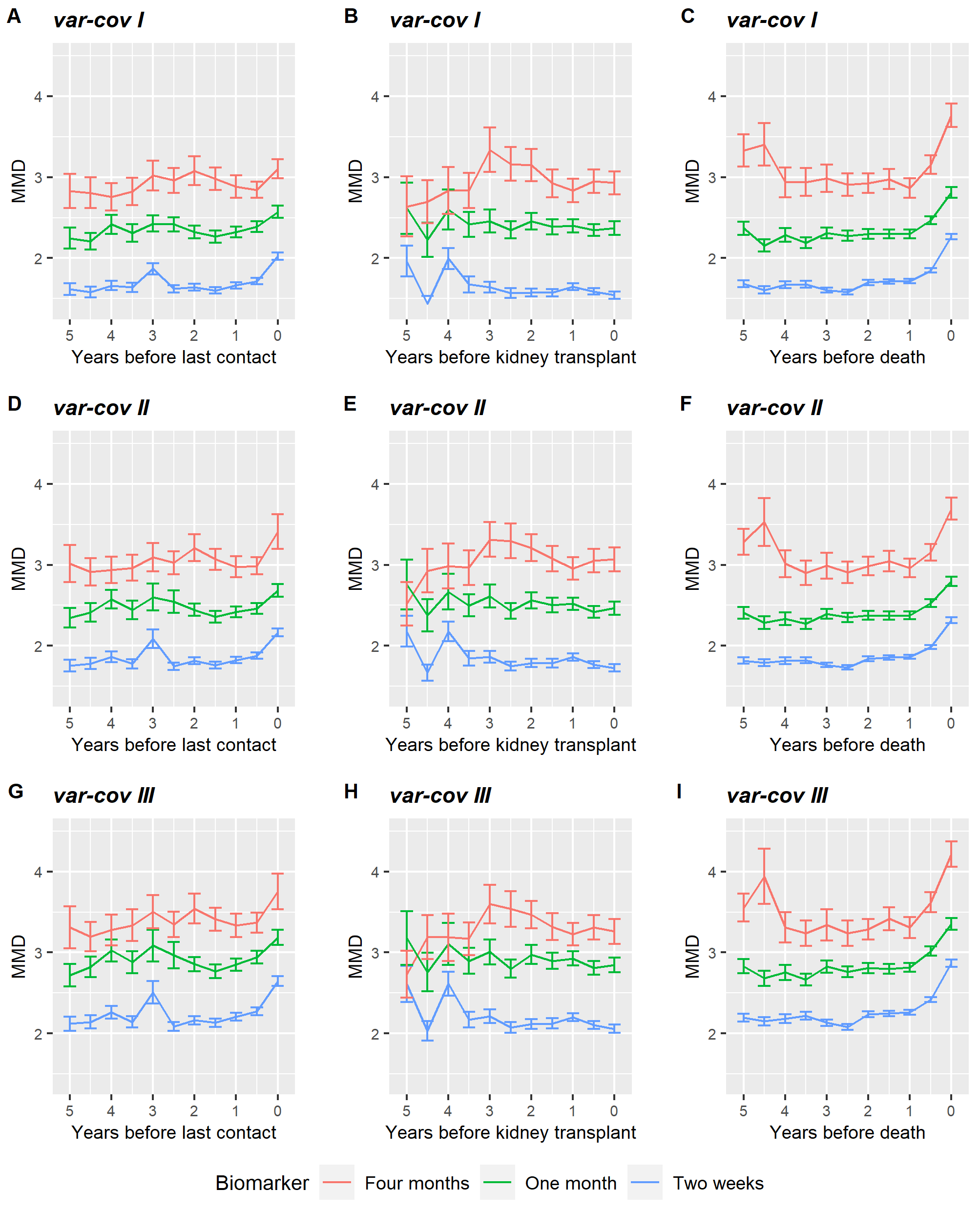


**Figure S7 Results are not sensitive to the precise variance-covariance matrix.** Panels A, B, and C show results using *var-cov* (I) (identity matrix, covariances = 0, equivalent to Euclidean space). Panels D, E, and F show results using *var-cov* (II) (based on samples within three months of death). Panels G, H, and I show results using *var-cov* (III) (based on samples at least two years before death). Panels A, D, and G are with censored (I) individuals (all loss to follow-up). Panels B, E, and H are with censored (II) individuals (those with a kidney transplant). Panels C, F, and I are with deceased individuals, excluding those with missing biomarker profiles in the 30 days preceding death. All the individuals above were from the "Full" dataset
